# Bottlenecks and opportunities for synthetic biology biosafety standards

**DOI:** 10.1038/s41467-022-29889-y

**Published:** 2022-04-21

**Authors:** Lei Pei, Michele Garfinkel, Markus Schmidt

**Affiliations:** 1 Biofaction KG, Vienna, Austria; 2 EMBO Policy Programme, EMBO, Heidelberg, Germany

**Keywords:** Synthetic biology, Environmental biotechnology, Bioremediation

## Abstract

The lack of innovative standards for biosafety in synthetic biology is an unresolved policy gap that limits many potential applications in synthetic biology. We argue that a massive support for standardization in biosafety is required for synthetic biology to flourish.

## Standards in synthetic biology

As synthetic biology aims to make biology easy to engineer, and to make it a real engineering discipline (as, for example, electronic engineering), synthetic biologists have called for the establishment of synthetic biology standards^1–3^. Unlike well-defined and standardized electronic parts, we still lack precisely defined genetic parts and proper standards. While some standards have been set for biology (such as standards for plasmids, for genetic circuits, and for enzyme research) much more needs to be done (see Box 1. for more detail).

Box 1 Examples for some (synthetic) biology standards **Standardization of plasmid: SEVA system**

SEVA stands for Standard European Vector Architecture. It is a web-based open repository of plasmid vectors for prokaryotes^9^. The SEVA plasmid repository has implemented a standard for physical assembly of vector plasmids and for their non-ambiguous nomenclature.

**Standardization of genetic parts: BioBricks and iGEM**

BioBrick parts are interchangeable DNA sequences with defined biological functions, which can be used to build new biological circuits by combining different parts together. The international Genetically Engineered Machine (iGEM) competition, for example, uses a registry of standard biological parts that follow the BioBricks assembly standard^36^. iGEM can been seen as a testbed for standardization in synthetic biology^37^, biosafety^10,38^, and risk management^39^.

**Standardization of enzyme: STRENDA**

STRENDA has set standards for data reporting in enzyme research to improve the quality of enzyme related data in scientific publications^40^.

**Standardization in screening synthetic genes and customers**

The International Gene Synthesis Consortium^41^ has adopted a standard protocol for screening both the sequences of synthetic gene orders and the customers who place them^42^. While originally intended to safeguard biosecurity it also has a biosafety function since it restricts access to specific DNA from regulated pathogens and to individuals and institutions that are permitted to handle such pathogens safely and securely.

## Why does biosafety need standardization?

Thinking about safety with respect to biological agents broadly, biosecurity frequently comes first to mind. And this is understandable, given that biosecurity as a field aims to prevent intended harm to people or the environment. Biosafety, in contrast, is a field focused on “containment principles, technologies and practices that are implemented to prevent unintentional exposure to biological agents or their inadvertent release”, as defined by the WHO^4^.

Biosafety guidelines are composed of policies, rules, and procedures to handle microorganisms and microbiological products. Implementing these biosafety guidelines requires suitable infrastructures (lab design and facilities), proper personal protection equipment, and sufficient staff training and surveillance (collectively called *biocontainment* as defined by the US Department of Health and Human Services)^5^. A key challenge, however, is extending the concept of biocontainment. In addition to the physical and design features noted in many definitions, containment that is engineered into the organism and that provides specific safety features after an (intentional) environmental release (e.g., restriction of horizontal gene flow, auxotrophy) is now included as well in biocontainment definitions^6–11^. The purpose of biocontainment, whether by laboratory or equipment design, or by constructing organisms intended for release, is identical in both cases: essentially, reducing potential hazards.

The growing research on engineering microorganisms, especially by practitioners in the area of synthetic biology, for industrial, medical, and environmental applications brings up new challenges for biosafety. There have been studies dedicated to addressing these challenges by developing new approaches to confine the engineered organisms to reduce the risks of unintentional exposure or to limit horizontal gene transfer for those organisms that would be released into the environment for medical or environmental applications^10,12^. Meanwhile, in addition to technical solutions to enhance biosafety, the research community has proposed other solutions, such as promoting the concept of ‘safety by design’ in biological studies^13^, and promoting a biosafety-focused culture^14^.

The goal to define standards for synthetic biology also includes standards for biosafety, as real-world applications need not only to be effective and, preferably, inexpensive, but obviously also “safe enough”. Setting up comprehensive biosafety standards is a critical prerequisite (although not a sufficient one) for synthetic biology products and processes to successfully go through a regulatory risk assessment and be approved for the market. From an industrial and regulatory perspective, proper deployment of biosafety standards could make it easier to conduct the risk assessment. Compared to conventional environmental risk assessment, a qualified presumption of the safety of an organism could even be granted a fast track evaluation^15^.

## What is the bottleneck for synthetic biology biosafety standards?

With the relevance of synthetic biology biosafety standards clearly visible, we wondered why there is such a lack of robust, standardized, and implemented biocontainment strategies. Is it because there are no technical solutions in the pipeline, or is it because existing promising solutions (which we may call proto-standards: technical solutions that could potentially become bona fide standards in the future) have not yet risen to this level? We set out to find evidence that either one or both of these gaps explain the somewhat puzzling lack of biocontainment standards. By identifying the key gap, we would be better placed to understand how to channel future efforts in the most effective way.

Searching peer-reviewed publications in Pubmed (keywords: containment, biosafety, synthetic biology, genetic engineering, CRISPR, gene flow, safeguard, kill switch, genetic code engineering, auxotrophy, cell free, chromosome free), we found 53 biosafety and biocontainment proto-standards with a potential to be applied in synthetic biology. Thematically the proto-standards are quite diverse, ranging from physical containment^16^ to synthetic auxotrophy^17–19^, kill switches^20,21^, semantic biocontainment such as genetic code engineering^22–25^, CRISPR safeguards such as gene drives^26,27^, DNA barcoding^15,28^, and chromosome free systems^29^. Underlying all of these is an implied reliance on relevant metrics: what do we measure to assure the proto-standards are working as described, and what metrics are required to know when a proto-standard can become a standard?

Despite several review articles that provide good overviews of the strategies developed for biocontainment purposes^10–12,30–33^ as far as we know, the list we put together represents the most comprehensive collection of biosafety and biocontainment solutions. Thus, we decided to structure the information, including its main feature, the microorganisms involved; its efficiency (measured or estimated by escape frequency), the tested or proposed application(s), the concern(s) or constraint(s), and the reference to make it easily usable and publicly available as an online Biocontainment Finder at https://standardsinsynbio.eu/biocontainment-finder/.

## Policy perspectives for biosafety standardization

The entries in the Biocontainment Finder show that there is no real shortage of academic papers and proofs-of-concept of novel and often creative ways to implement biosafety at different levels in living cells. However, real-world applications with these biocontainment principles applied are still missing. The critical shortcoming is the translation of academic findings and proof-of-principle to a bona fide engineering level that would make it of interest to industry. Very few examples go as far as reporting the escape frequency or other relevant metrics^14^ to evaluate the usefulness and validity of the biocontainment approaches. There is an apparent gap between academic research and useful applications.

We can compare this to a similar problem in medicine, where the gap from bench to bedside is seen as a critical bottleneck to improve the life and health of patients. The interdisciplinary approach of translational medicine was created to better connect bench and bedside to close this gap. One of the differences in biosafety standardization is that, unlike in translational medicine, there is currently no self-identified constituency that works cohesively to make it a reality, rather than just a good idea.

Instead, moving toward the greater use of standards in biosafety and particularly at the biosafety/synthetic biology intersection will require explicit or tacit agreements among the stakeholders who may not be natural or cohesive constituencies generally, but who all have the same goal: safety in research, for health and the environment. Beyond this, other goals (advancing research, achieving desired societal outcomes, wealth creation) may not be fully overlapping, but large swathes of them can certainly be achieved. In earlier work^34^ that included expert elicitation from many stakeholders, we have shown that six potentially non-overlapping or even conflicting goals can still be achieved, recognizing the need for the following actions:

1. *Clarify necessity for benchmarks, and for platforms for stakeholder discussions*. Industrial concerns about standards are often characterized as “negative”; i.e., industry would not want them as they would add additional burden to the production process, disrupt product diversity or company goals. While this may be true in some cases, in general, any tool that makes the development of applications more straightforward, less uncertain, and possibly eases regulatory processes could in principle be welcomed both by companies and by regulators. As well, diversity in products should be valued, but for regulatory approval (and public acceptance) regarding underlying technologies, the more standardized the process(es) leading to these are, the more likely they will be approved, or approved more easily. This will require ongoing discussions between stakeholder groups. It will be critical to form a platform that allows for very open discussions between these groups.
2. *Confirm importance of case-by-case oversight for regulators and for biosafety organizations in the context of defined cases.* While underlying standardization in product development should ease regulatory approvals (and thus at least tangentially public acceptance), it remains critical that regulators assert approvals for each case. What will be critical here is being able to recognize and define what a case is. For example, any instance of a new product could be a case (for example, for mRNA vaccines that vary even by just a few nucleotides) while for other types of products, perhaps tens or even hundreds could be approved as a single case.
3. *Recognize the need for researchers to test standards and to receive credit for that testing and for implementation*. In the assessment of researchers, drawing the line between what is an inherent part of research and what is tangential to or outside the research can be difficult to draw. And in some cases, even activities that are “outside” the research are still important for research and researchers. We see these discussions now most pointedly around open science and research integrity: should activities in those areas be rewarded directly, or are they simply assumed as baselines? Here, it is apparent that there is a necessity for rewarding work not only to build standards, but to assure they are distributed to and understood in the community. For some researchers, their organizations may already be changing their reward systems. For others, assuring such rewards may require systemic changes by their organizations. It will be critical for other stakeholders discussed here to help support the idea that not only developing standards but also testing and implementing them are worthy of reward.
4. *Expand roles for publishers.* Journals can play an important role not only in supporting researchers to be able to describe their work in the most useful way (addressing technical features of a paper that help the information in it to be reusable, interoperable, reproducible, or any stated goal), but by helping to define the scope of what is required in the necessity of the use of, or the reporting of a standard. In general, publishers will not demand anything that a particular research community itself does not stand behind. There will need to be discussions between the stakeholders discussed here to determine if there are any “absolutes” that should be required to publish a research paper. Those absolutes will of course vary by journal and by community.
5. *Implement safety standards in the do-it-yourself/do-it-together communities*. Interestingly, the communities of researchers functioning outside of classical research performing organizations have emphasized the need for (and desire to use) standards strongly and consistently^32,34,35^. While there are several possible explanations for this, at least part of this might be credited to the additional interest of this group in having regulations generally loosened (and particularly for DIY individuals and groups, who might sometimes be subject to additional regulation). By embracing standards as a way to ensure more consistency in biosafety, this community can quite reasonably then look for ways to loosen other oversight. As noted above, this would be true for all research communities. However, we particularly saw the enthusiasm for implementing safety standards in the DIY community in our earlier analysis^34^ and thus this would be an ideal group to pilot and report back on the use of these standards.
6. *Assure a framework for moving from “biosafety standards” to simply “standards” that would be part of synthetic biology standards generally*. The underlying motivation in this idea comes from an understanding of how standards organizations work: these organizations are responsible for managing the process of deliberating the need for and scope of a new standard. They are not responsible for inventing standards. Thus, while we can think of “biosafety standards” as a special case for some policy discussions, in fact, they are subject to the same processes as any development of standards. The community is responsible for assuring the standard is useful and is further responsible for assuring it is in fact used by the community. Because standards are, with few exceptions, voluntary, this means researchers, DIYers, industry, and other concerned stakeholders will need to do the hard work. As discussed above, sometimes such work is rewarded, sometimes it is not. It will thus be critical as part of training in research, particularly in the dynamic community identified as synthetic biology, that all participants understand the importance of standards, and individual’s possible roles in helping them come to fruition.

Thus, an important role for the synthetic biology community and particularly for those already convinced of the value of standards would be to take on some “science-and-policy experiments” in biocontainment or biosafety standardization. What this means is that it will take a subset of the community to commit to using biocontainment standards in their own work, reporting back to the rest of the community regarding the benefits or problems that arise in those approaches, and subsequently working to improve those standards. This is something that could happen with minimal incentive structures, such as funding networking activities between academic and industrial researchers, and thus strengthen the possibility of achieving some universal biocontainment standards.

## Outlook

The issue of biosafety standardization in synthetic biology has important implications at both the technical and policy levels. For the technical perspective, there is no shortage of existing and novel biocontainment strategies spanning physical, chemical and biological types of containment, ranging from well-documented, all the way to proof-of-concept tools. What is notable in reviewing this range is the surprising diversity and ingenuity of novel biosafety solutions. And although the conceptual tools are of course not yet standards, they allow the community to test and select specific technical solutions, thus laying the foundation for community approved novel biosafety standards.

From a policy perspective, the wealth of identified biosafety and biocontainment solutions provides clear coordinates for constructive intervention. The set of biosafety entries describe the laboratory side that defines the gap between academic research and real-world applications. In concert with the use of suitable metrics, we can foresee an effective, focused and measurable way forward by providing the means to carefully analyze and select promising biocontainment candidates from this set for further development and to improve their technology readiness levels.
